# Correction: Ding et al. Recent Trends in Foliar Nanofertilizers: A Review. *Nanomaterials* 2023, *13*, 2906

**DOI:** 10.3390/nano16020114

**Published:** 2026-01-15

**Authors:** Yanru Ding, Weichen Zhao, Guikai Zhu, Quanlong Wang, Peng Zhang, Yukui Rui

**Affiliations:** 1 College of Resources and Environmental Sciences, China Agricultural University, Beijing 100193, China; dingyr314@163.com (Y.D.);; 2 Department of Environmental Science and Engineering, University of Science and Technology of China, Hefei 230026, China

In the original publication [1], reference 52 was retracted prior to publication. Concerns were raised regarding this matter, and the reference in question was removed from the reference list. The content cited in Table 1 was also removed.

“52. Jafari, A.; Hatami, M. Foliar-applied nanoscale zero-valent iron (nZVI) and iron oxide (Fe_3_O_4_) induce differential responses in growth, physiology, antioxidative defense and biochemical indices in *Leonurus cardiaca* L. *Environ*. *Res*. **2022**, *215*, 114254.”

With this correction, the order of some references has been adjusted accordingly. The authors state that the scientific conclusions are unaffected. This correction was approved by the Academic Editor. The original publication has also been updated.

## References

[1] Ding Y., Zhao W., Zhu G., Wang Q., Zhang P., Rui Y. (2023). Recent Trends in Foliar Nanofertilizers: A Review. Nanomaterials.

